# Clay illuviation provides a long-term sink for C sequestration in subsoils

**DOI:** 10.1038/srep45635

**Published:** 2017-04-06

**Authors:** Gemma Torres-Sallan, Rogier P. O. Schulte, Gary J. Lanigan, Kenneth A. Byrne, Brian Reidy, Iolanda Simó, Johan Six, Rachel E. Creamer

**Affiliations:** 1 Teagasc, Johnstown Castle, Wexford, Republic of Ireland; 2 Department of Biological Sciences, School of Natural Sciences, University of Limerick, Limerick, Republic of Ireland; 3 Farming Systems Ecology, Wageningen University and Research, Wageningen, The Netherlands; 4 Department of Environmental Systems Science, Swiss Federal Institute of Technology, ETH Zurich, Zurich, Switzerland; 5 Soil Biology and Biological Soil Quality, Wageningen University and Research, Wageningen, The Netherlands

## Abstract

Soil plays a key role in the global carbon (C) cycle. Most current assessments of SOC stocks and the guidelines given by Intergovernmental Panel on Climate Change (IPCC) focus on the top 30 cm of soil. Our research shows that, when considering only total quantities, most of the SOC stocks are found in this top layer. However, not all forms of SOC are equally valuable as long-term stable stores of carbon: the majority of SOC is available for mineralisation and can potentially be re-emitted to the atmosphere. SOC associated with micro-aggregates and silt plus clay fractions is more stable and therefore represents a long-term carbon store. Our research shows that most of this stable carbon is located at depths below 30 cm (42% of subsoil SOC is located in microaggregates and silt and clay, compared to 16% in the topsoil), specifically in soils that are subject to clay illuviation. This has implications for land management decisions in temperate grassland regions, defining the trade-offs between primary productivity and C emissions in clay-illuviated soils, as a result of drainage. Therefore, climate smart land management should consider the balance between SOC stabilisation in topsoils for productivity versus sequestration in subsoils for climate mitigation.

The Intergovernmental Panel on Climate Change (IPCC) provides good practice guidance on the methodology to account for the impact of land use management and land use change on organic carbon stocks in soils^1^. Generally for Tier 1 (default) or Tier 2 (national-specific) approaches, a stock change is calculated by assessing the reference soil organic carbon (SOC) level down to 30 cm and measuring again after a period of at least 3 to 5 years^1^. However, this approach neither accounts for different turnover rates of different fractions of SOC, nor recognises that substantial pools of recalcitrant SOC which exist below 30 cm, and may be affected by land management. For example, artificial drainage of intensive grassland systems is a common practice in many temperate countries to increase the grazing capacity of soils and extend the grazing season. The application of artificial drainage systems has been found to significantly affect the SOC recalcitrance in soils^2^,^3^. However, very few studies exist in grassland systems where empirical data is available for SOC fractions below 30 cm^4^,^5^.

To resolve this issue, sophisticated biogeochemical cycling models are commonly applied to extrapolate SOC pools and recalcitrance to estimate potential source/sink reservoirs at lower depths. These include; the RothC carbon model^6^, CENTURY model^7^, and the DeNitrificationDeComposition (DNDC) model^8^, which all divide SOC inputs into easily decomposable and recalcitrant pools, which are subsequently input into labile, recalcitrant and inert/passive SOC pools following mineralisation. These models include the simulation of physical protection via the clay fraction, which diverts SOC flows to the humic fraction rather than CO_2_ mineralisation^9^. However, these pools are usually defined kinetically and in the absence of empirical data on the mean residence times associated with the different chemical and physical properties^10^.

There have been attempts to directly relate these modelled pools to the different aggregate fractions^11^,^12^,^13^. Aggregation occurs when particulate organic matter (POM) starts to be decomposed. Bacteria mineralize POM, generating a mucilage that serves as an adhesive between the mineral particles of the soil and the POM^14^. Through the encrustation of POM with mineral particles, SOC becomes enclosed into large macroaggregates (2–8 mm) and small macroaggregates (250–2000 μm). As POM undergoes further decomposition, it decreases in size and gets encapsulated into microaggregates within macroaggregates^15^. This structure may weaken and break, creating free microaggregates (53–250 μm) (Figure SI1). Soil organic carbon also binds chemically to clay minerals, the smallest particles in soils (<20 μm), which due to their large surface area, have the capacity to adsorb high amounts of SOC, effectively protecting it against microbial decomposition through different binding mechanisms^16^,^17^,^18^.

The fractionation process assumes that SOC associated with microaggregates is older and more stable than SOC contained in macroaggregates, with a much slower turnover time^19^,^20^,^21^,^22^,^23^,^24^,^25^,^26^. The turnover time of SOC contained in macroaggregates is reported as 1 to 10 years, while SOC associated with microaggregates is considered stable up to 100 years in soil. Silt plus clay associated SOC (<53 μm) is stable in excess of 100 years^21^. Therefore, encapsulation of SOC within microaggregates or association of SOC with silt plus clay particles is considered long-term C storage^12^,^27^,^28^.

Our research aimed to elucidate the patterns of SOC distribution in typical temperate grasslands in Ireland (Table SI2), and particularly the following two key research questions: (1) Does the topsoil and subsoil have similar physical protection against mineralisation? (2) How does this vary across soil types, specifically between freely draining soils and those subject to clay illuviation over depth?

## Results

### SOC distribution within the soil profile

**Figure 1:**
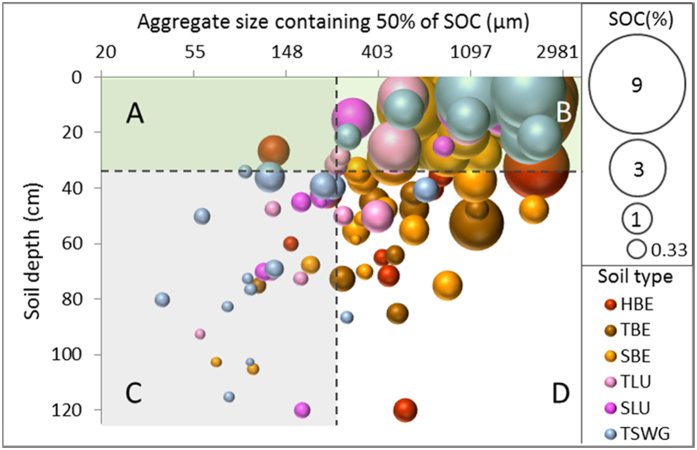
Relative distribution of SOC within aggregates by depth. The figure plots the aggregate size containing 50% of SOC on a logarithmic scale (x-axis). The y-axis is the soil depth (cm). The figure is divided in four quadrants: (**A,B**) (top) indicate the top 30 cm of all soil profiles, while quadrants (**C,D**), (bottom) indicate the subsoil. Quadrants (**A,C**) (left) correspond to the samples where more than 50% of SOC is located in silt plus clay and microaggregate fractions – indicating stability as C associated with smaller aggregates is more protected against mineralisation. Quadrants (**B,D**) (right) show the samples that reach 50% of SOC only when the macroaggregates or large macroaggregates are taken into account. Bubble size represents total SOC of the bulk sample at that depth. Colours indicate individual soil subgroups: HBE = Humic Brown Earth; TBC = Typical Brown Earth; SBE = Stagnic Brown Earth; TLu = Typical Luvisol; SLu = Stagnic Luvisol; TSWG = Typical Surface-water Gley.

The distribution of SOC across the four different aggregate size classes in all six soil types (Fig. 1) reveals three patterns:

1. The total amount of SOC declines with depth in all soils;
2. The proportion of SOC associated with large and small macroaggregates declines with depth in all soils;
3. A larger proportion of the SOC is associated with microaggregates and silt plus clay fractions in soils affected by clay illuviation (namely: Typical Luvisols (TLu), Stagnic Luvisols (SLu) and Typical Surface-water Gleys (TSWG)) than in Brown Earths (Humic Brown Earths (HBE), Typical Brown Earths (TBE) and Stagnic Brown Earths (SBE)).

As a result, most (68.9% ± 11.5) of the SOC was found within the top 30 cm of the soil profiles, and a significant proportion (84% ± 9.5) of this topsoil SOC was located within large and small macroaggregates, as indicated by the predominance of the ‘large bubbles’ in quadrant B.

A smaller proportion (16.1 ± 9.1%) of the SOC in the top 30 cm was associated with the microaggregates and silt plus clay, as indicated by the ‘almost empty’ quadrant A. In contrast, SOC associated with the smaller fractions in the subsoil (30 cm to 1 m depth) equates to 42.2 ± 19.5% of SOC stock (Quadrant C).

### Differences in SOC distribution between soil types

Below 30 cm, differences between soil types emerge: quadrant C shows that in the subsoils affected by clay illuviation (namely: Typical Luvisol, Stagnic Luvisol and Typical Surface-Water Gley), where more than 50% of the SOC is associated with microaggregates and silt plus clay fractions. At the same time, quadrant D shows that this is not the case for the Brown Earths (Humic Brown Earth, Typical Brown Earth, Stagnic Brown Earth), where the proportion of SOC in microaggregates and silt plus clay fractions increases with depth but the majority of SOC remains associated with large and small macroaggregates, even at depth.

The differences in SOC distribution between free-draining Brown Earths (Humic Brown Earth, Typical Brown Earth, Stagnic Brown Earth) (Cambisols, WRB, 2006) and soils subject to clay illuviation (Typical Luvisol, Stagnic Luvisol and Typical Surface-Water Gley) (Stagnosols and Luvisols, WRB, 2006) are significantly different, however, no significant differences were observed between individual soil types within these two categories (Table 1).

## Discussion

The soils used in this study are important soils for livestock production systems in temperate grassland regions. For example, Typical Brown Earths, Typical Luvisols and Typical Surface-water Gleys account for 762 k, 448 k and 311 k hectares of the agricultural land area in Ireland, respectively, which equates to 47.9% of the total grassland area (3176 k hectares). Whilst the total quantity of SOC stocks of these soils are similar (Table SI2), the results from our fractionation show that the stability of the SOC stocks do vary significantly between soil types, specifically soils that are subject to clay-illuviation versus free-draining Brown Earths. This has significant implications both for soil organic carbon storage over the long term and land-use management of these soils, not just in an Irish context, but worldwide, as these soils are often the dominant agricultural soils of many grassland and tillage regions of the world^29^.

In this context, our results indicate that it is important to consider soil depth and SOC distribution within aggregates when measuring and/or modelling the SOC sequestration potential of soils in grassland systems. This finding concurs with several studies that highlight the importance of identifying the mechanistic relationship between aggregates and SOC, in order to have a better understanding of long term SOC storage mechanisms in soils and therefore potential for C sequestration^19^,^20^,^22^,^30^.

A sampling depth of 30 cm has been frequently applied to quantify the stock of SOC and the associated quality of that stock through fractionation studies. In this study, we found that sampling to a depth of 30 cm only captures 69% of the SOC located within the first meter, this is comparable to other studies in this field^31^,^32^. In addition, we clearly demonstrate that the majority of this topsoil carbon is associated with macro aggregates, on all grassland soils and is therefore a mineralisable resource.

Macroaggregates are formed around particulate organic matter (POM), which is derived from the decomposition of fresh residues^15^,^33^. We suggest that within the topsoil (0–30 cm), the process of aggregate formation is primarily influenced by vegetation type and root exudates, which is in line with other studies suggesting that topsoil SOC characteristics mainly reflects the vegetation type^34^,^35^. Distribution of SOC within aggregates in the topsoil is similar between soil types given that vegetation type and SOC stocks of the rhizosphere are quite consistent across the studied grasslands.

At lower depths (below 30 cm depth) in the soil profile, SOC is increasingly associated with smaller aggregate sizes. Here, differences between soils become more pronounced: in Brown Earths, the majority of SOC is still associated with macro-aggregates, while in soils subject to clay-illuviation, the majority is associated with micro aggregates and silt plus clay fractions. In the subsoil mineral properties, such as clay content and chemical composition, play a stronger role in the aggregation process and exert a much stronger influence on the SOC sequestration process^36^.

We also found that sampling to 30 cm causes a bias in the quantification of SOC sequestration potential of these soils. If we only consider the surface 30 cm of a soil profile, up to 84% of the SOC is less protected from mineralisation, as it is located in larger aggregate fractions. Hence, while the most dynamic fraction of the soil is well represented, the most stable fraction is remarkably underrated. This finding highlights the importance of sampling deeper soil horizons, which concurs with other recent studies^34^,^37^,^38^,^39^. This study also highlights that measuring only total SOC content alone does not reflect the stability of carbon in these soils but rather only the SOC stocks. SOC stocks can be similar between soil types, but it is the distribution of the SOC across aggregate size classes that determines how stable the SOC is at a given depth^15^,^40^ and therefore considered C sequestration. An illustrative example of this is the higher total values of SOC of Humic Brown Earth as compared to Stagnic Luvisol throughout the profile (Table SI1). Nevertheless, below 30 cm, SOC is located mainly in the large aggregate fractions in Humic Brown Earth, while in Stagnic Luvisol SOC is mainly associated with smaller fractions. Given that SOC occluded in macroaggregates has a turnover time of 1 to 10 years, as compared to the 10 to 100 years, and >100 years for SOC in microaggregates and silt plus clay fractions, respectively^21^. Therefore a Stagnic Luvisol has a greater degree of protection against mineralisation (long-term C storage) of its SOC compared to a Humic Brown Earth.

Many temperate grassland clay-illuviated soils are associated with poor or imperfect drainage due to the higher clay content with depth, causing stagnation of water percolating through the soil profile. The abolition of the EU milk quota in 2015 has resulted in significant investment in agricultural management practices with a projected increase in production from the dairy sector of 50% by 2020^41^. Management practices include extended grazing seasons and increased grass utilisation. To achieve this, there is a renewed emphasis on the installation of arterial drainage systems in imperfectly and poorly drained soils under grassland production. O’Sullivan *et al*.^42^ assessed the financial trade-off between drainage for increased primary production and carbon sequestration in grassland soils, exploring a range of hypothetical carbon prices for SOC. They highlighted that at current carbon prices there was no incentive for farmers to maintain current SOC stocks. EU and national policies highlight the importance of SOC sequestration in mitigation of climate change. At the recent meeting of the “@Agreement at the Convention of the Parties” (COP) 21 in December 2015, the French Government put forward the *4 per 1000 initiative* (http://4p1000.org/understand) which proposes soil management options for the sequestration and preservation of SOC and yet no support is currently available to land managers to not drain land in order to preserve the long-term storage of SOC sequestered at depth in the clay-illuviated soils. This paper summarises that for the long-term storage and preservation of sequestered carbon, we must also look to the potential of the sub-soil compartment and the soil management practices which influence this part of the soil profile.

## Conclusions

The current IPCC guidelines account for SOC stocks in the top 30 cm of soil. While this may adequately reflect the magnitude of soil carbon sinks, this fails to capture differences in the quality of these sinks, expressed in terms of the stability and hence residence time of the SOC.

While some models, such as RothC, CENTURY, and DNDC already simulate SOC pools with different turnover times as a function of the clay fraction of the topsoil, these do not consider changes in clay content with depth below 30 cm, which may significantly affect the stability of SOC or vertical re-distribution of dissolved C, specifically in soils subject to clay illuviation

Land management practices, such as the installation of arterial drainage systems on these latter soils, have a significant influence on the long-term stability of sequestered SOC at depth. In these soils, drainage for increased productivity may reduce the capacity for longer-term storage of SOC. Incentives to maintain these stocks do not presently exist and should be considered in the formulation of agricultural policies.

## Materials and Methods

### Soil selection and sampling

Thirty one grassland sites were sampled, representing six different soil types. Table SI 1 describes the range in soil types sampled and the correlation to the World Reference Base (WRB, 2006) classification system^43^. These soil types represent a range of SOC and textural characteristics typical of grassland soils occurring in Ireland (Table SI1).

At each site (see location of sites in Figure SI3) a profile pit was dug to a depth of 1 m, where possible. All horizons were described according to the FAO field handbook *Guidelines for soil description*^44^ and classified by means of the Irish Soil Information System^45^. A 1 kg sample was taken from the centre of each horizon and immediately stored at 4 °C, until use. Following the coning and quartering technique^46^, a 300 g subsample was sieved at 8 mm and dried at 40 °C for 7 days. Samples for soil bulk density measurement were taken in triplicate using (5 × 5 cm) cores from each horizon. See Table SI2 for chemical and physical information of each horizon.

### Aggregate separation

An adaptation of the wet sieving method^47^ was followed to separate each sample into four aggregate sizes: large macroaggregates (2–8 mm), small macroaggregates (250 μm−2 mm), microaggregates (53–250 μm) and silt plus clay (<53 μm) (Figure SI2). An 80 g subsample was placed on the top of a 2 mm sieve and submerged in distilled water for five minutes. This causes a slaking of the aggregates as the water enters into the pores, increasing the pressure and breaking the less stable aggregates. Subsequently, the sieve was manually moved up and down for a three minute period at a rate of 33 movements per minute, in order to make it a constant movement. Large macroaggregates and stones were retained on the 2 mm sieve. The material that passed through the sieve was further separated at 250 μm and 53 μm using a modification of the Eijkelkamp wet sieving apparatus.

The modified wet sieving apparatus was designed to fit four sieves of 10 mm diameter, to facilitate an overall sample size of 80–100 g of <2 mm sieved soil. Soil was placed on the top of the 250 μm sieves and moved up and down in water for a period of three minutes. The suspension collected through the sieve was then added to a 53 μm sieve and the process repeated. All material remaining on each sieve was washed into plastic containers, dried at 50 °C, weighed and ball-milled.

For each sample, the proportion of stones (>2 mm), coarse sand (250–2000 μm) and fine sand (53–250 μm) was analysed with a modification of the ISO 11277: 1998 for particle size analysis. The percentage of each aggregate size was calculated by subtracting same-sized sand content from the total fraction weight of each fraction.

### Soil Organic Carbon analysis

For each aggregate fraction carbonates were removed by acid fumigation, following the method described by Harris *et al*.^47^. SOC of each fraction was analysed with a LECO Truspec CN analyser following the ISO 10694:1195, and expressed on a sand-free basis^20^. To calculate the bulk soil SOC proportions throughout the profile, total SOC content of each horizon was multiplied by the bulk density of that horizon, thus obtaining g SOC cm^−3^. Since the data was collected on a per horizon basis, the weighted average of the SOC content of the different horizons was used to calculate the proportion of SOC in the first 30 cm.

### Statistical analysis

**Figure 2:**
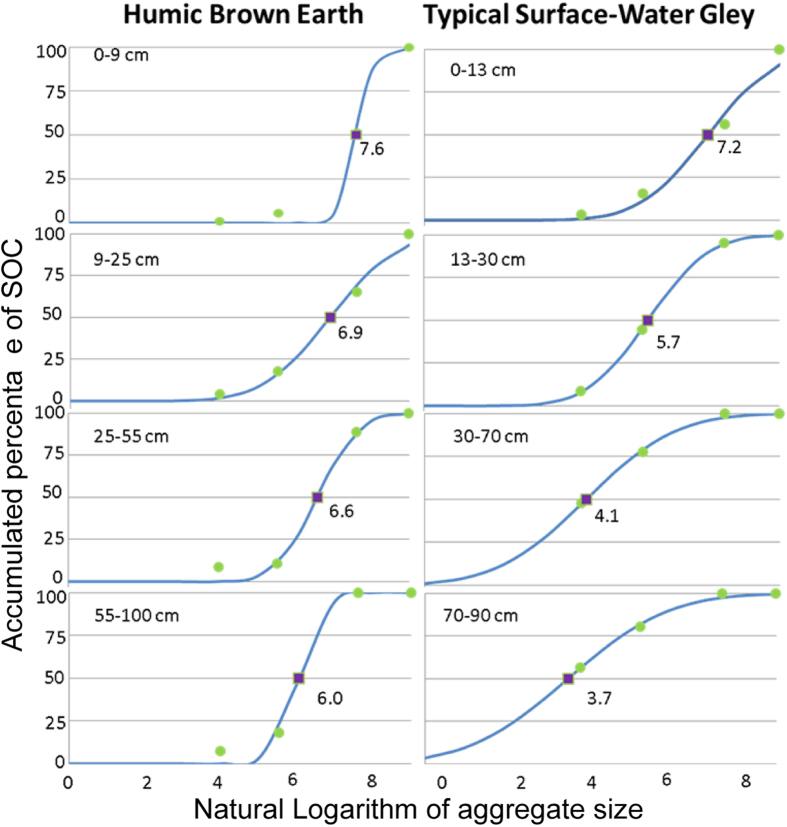
Example of *m* value for two soils at depth. The square (*m*) is the point of the fitted curve where 50% of the SOC is reached. Green dots represent the actual observations. The blue line is the fitted curve.

Analysis was performed at horizon level for each soil profile described. The physical fractionation process resulted in four relative aggregate size proportions for each horizon and four associated SOC contents. The relative distribution of SOC associated with each fraction was calculated by multiplying the proportion of each of the fractions by its SOC content, and dividing the result by the total amount of SOC in that sample. In order to synthesise the data, we summarised the distribution of SOC across the aggregate sizes by applying a normal distribution to each sample against the log-transformed aggregate size, and deriving the *m*-statistic, which is the mean of the distribution, and thus the natural logarithm of the aggregate size that cumulatively contains 50% of the SOC in the sample, starting from the silt plus clay fraction and progressively including the SOC located in the bigger size fractions (See Fig. 2). When *m* is smaller than 5.5 more than 50% of the SOC at that depth is located in the silt plus clay and microaggregate fractions, given that 5.5 is the natural logarithm of the upper boundary of microaggregate size class (250 μm). In the example presented in Fig. 2, the decrease of *m* in deeper horizons indicates that most of the SOC is associated with smaller aggregate size classes in deeper soil layers. It can then be observed that, in the case of Typical Surface-water Gley, *m* is decreasing markedly at depth, in contrast with Humic Brown Earth. Hence, *m* is a good tool to distinguish between soil types.

Backwards regression was performed to assess the dependence of *m* on depth, soil type and interaction between them.

## Additional Information

**How to cite this article:** Torres-Sallan, G. *et al*. Clay illuviation provides a long-term sink for C sequestration in subsoils. *Sci. Rep.* **7**, 45635; doi: 10.1038/srep45635 (2017).

**Publisher's note:** Springer Nature remains neutral with regard to jurisdictional claims in published maps and institutional affiliations.

## Supplementary Material

Supplementary Information

## Figures and Tables

**Table 1:** Multiple linear regression of soil properties on ‘m’ across all sites and depths.

Explanatory variables	Soil type used as the baseline in the backward regression											
	TSWG		SLu		TLu		SBE		TBE		HBE	
Intercept	6.83		6.83		7.00		7.51		7.27		7.27	
Depth	−0.0244		−0.0244		−0.0271		−0.0246		−0.0195		−0.0195	
	**main effect**	**xdepth**	**main effect**	**xdepth**	**main effect**	**xdepth**	**main effect**	**xdepth**	**main effect**	**xdepth**	**main effect**	**xdepth**
TSWG			n.s.	n.s.	n.s.	n.s.	−0.652	n.s.	n.s.	−0.0118	n.s.	−0.0118
SLu	n.s.	n.s.			n.s.	n.s.	−0.608	n.s.	−0.586	n.s.	−0.586	n.s.
TLu	n.s.	n.s.	n.s.	n.s.			−0.728	n.s.	n.s.	−0.0159	n.s.	−0.0159
SBE	0.600	n.s.	0.600	n.s.	0.543	n.s.			n.s.	n.s.	n.s.	n.s.
TBE	0.676	n.s.	0.676	n.s.	0.608	n.s.	n.s.	n.s.			n.s.	n.s.
HBE	0.742	n.s.	0.742	n.s.	n.s.	0.0156	n.s.	n.s.	n.s.	n.s.		

Coefficients noted “n.s.” were not significantly different from 0 (p < 0.05) and thus progressively removed from the model. The main effect of depth was significant for all soil types.
