# Supplementary material for: Clay illuviation provides a long-term sink for C sequestration in subsoils
Source: Sci Rep. 2017 Apr 6;7:45635. doi: 10.1038/srep45635 (PMC5382536; doi:10.1038/srep45635)

Clay illuviation provides a long-term sink for C sequestration in subsoils.

Torres-Sallan, G., Schulte, R.P.O, Lanigan, G.J., Byrne, K.A., Reidy, B., Simo, I., Six, J., Creamer, R.E.

Supplementary Information

Figure SI1. Soil aggregate structure diagram

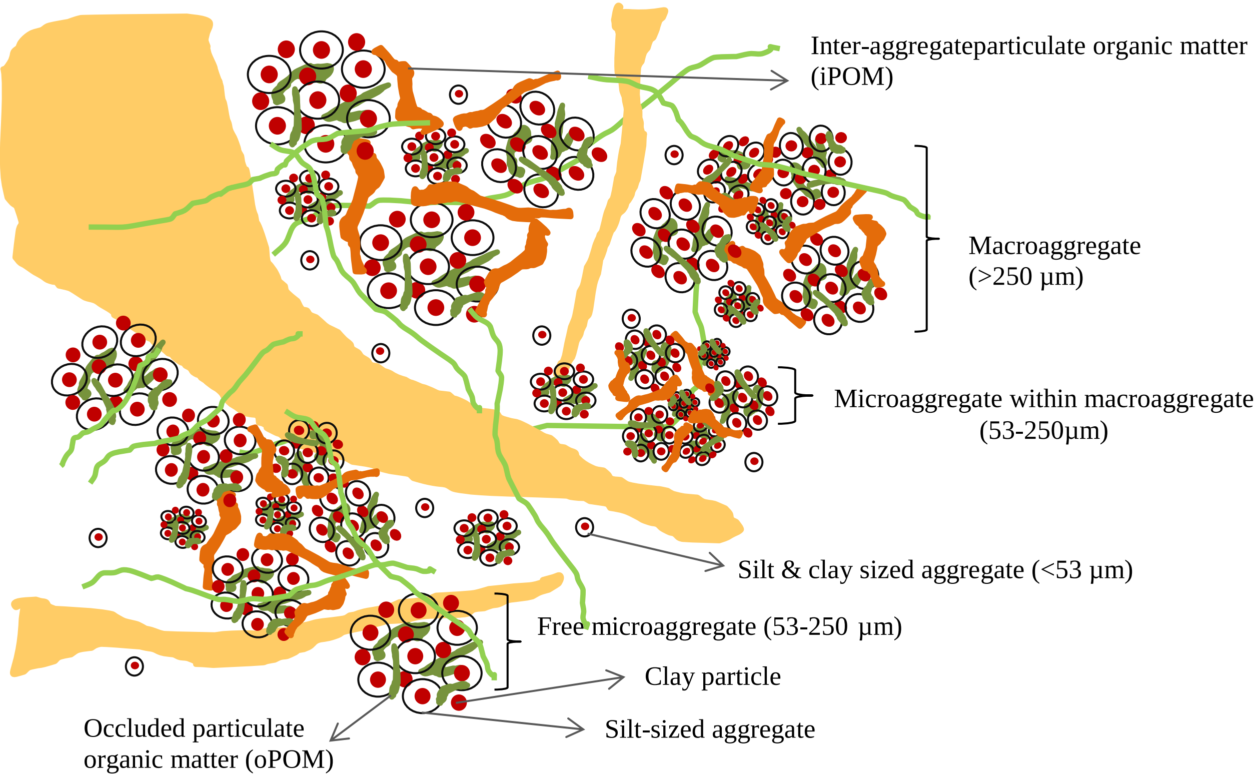

Table SI1. Description of soil subgroups distribution and characteristics in Ireland.

Soil Type	WRB Correlation	Diagnostic Description	Coverage of grassland land area (ha)	Indicative SOC to 1 m depth ($t\cdot ha^{-1}$)
Typical Brown Earth (TBE)	HaplicCambisol	Well drained soils.	761,944 ha	126.96 +/- 33.5
Humic Brown Earth (HBE)	HumicCambisol	Well drained soils with a humose topsoil.	43,139 ha	124.43 +/- 30.9
Stagnic Brown Earth (SBE)	StagnicCambisol	Moderately drained soils with a slowly permeable sub-surface horizon.	107,101 ha	121.98 +/- 37.5
Typical Luvisol (TLu)	HaplicLuvisol	Moderately drained soils due to clay illuviation, reducing permeability in the sub-surface horizon	448,446 ha	170.31+/- 113.8
StagnicLuvisol (SLu)	StagnicLuvisol	Imperfectly drained soils due to clay illuviation and gleying below 40 cm, as a result of a slowly permeable sub-surface horizon.	140,864 ha	108.89 +/- 22.0
Typical Surface-water Gley (TSWG)	HaplicStagnosol	Poorly drained soils, with gleying within 40 cm, as a result of a slowly permeable sub-surface horizon.	311,201 ha	128.21 +/- 52.1

This information was synthesised from the Irish Soil Information System database and maps [41,45] .

Figure SI2

100 g subsample

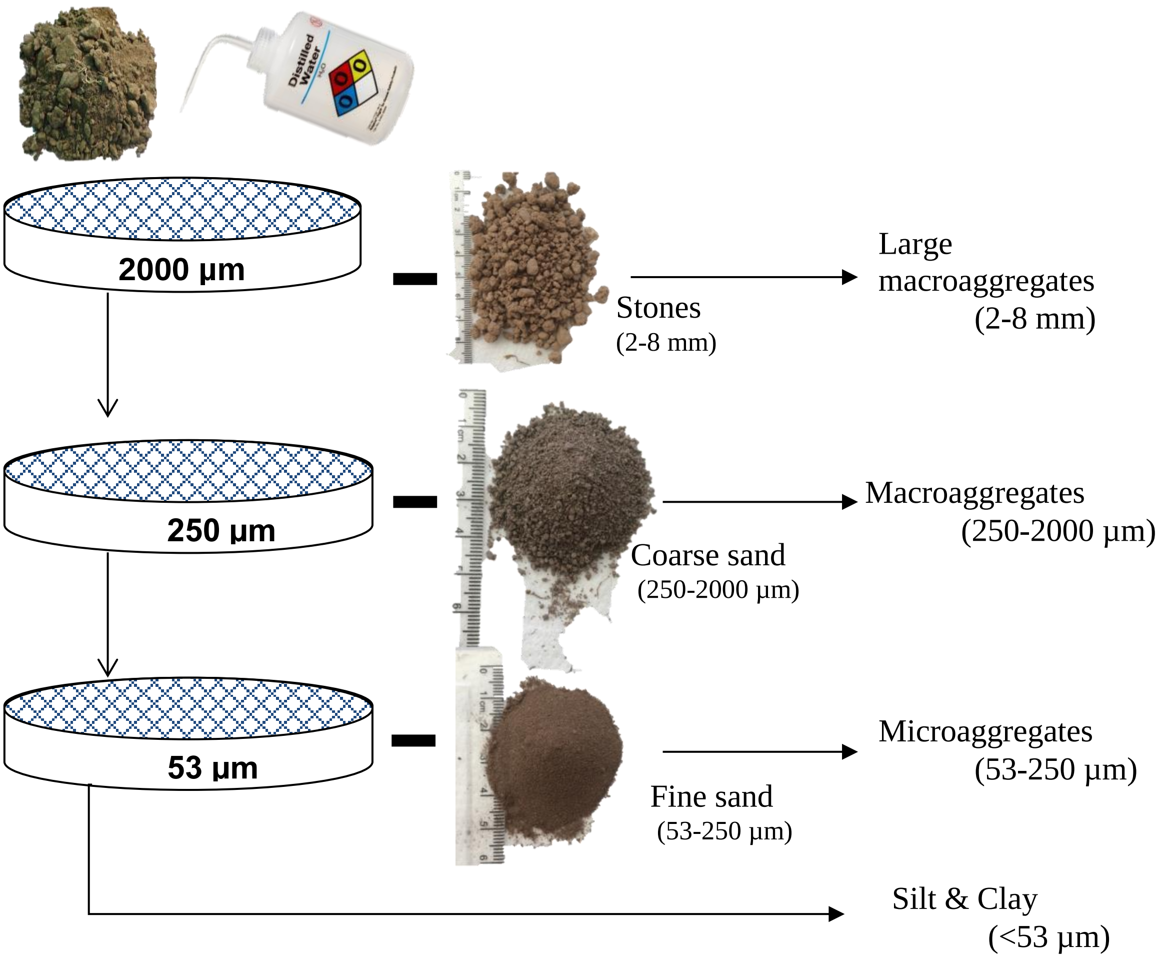

Method for aggregate fractionation adapted from Cambardella and Elliott, 1993

Table SI2. Soil chemical and physical properties of each sample. Note: End depth 999 indicates that the pit was not deep enough to arrive at the end of the last horizon. Source: Massey et al., 2014 [42]

Profile Number	Horizon	Soil type	Start depth	End depth	Base Saturation (%)	Cation Exchange Capacity (cmol kg^{-1})	N (%)	C (%)	Corg (%)	pH	Sand 200-005mm % w/w	Silt 0002 - 005mm % w/w	Clay <0002mm % w/w	Textural Class	Bulk density g/cm3
RC042	1	TBE	0	25	94	20.33	0.50	5.07	3.80	6.83	32	36	32	Clay Loam	0.83
RC042	2	TBE	25	70	96	6.53	0.16	1.52	0.79	7.04	57	32	11	Sandy Loam	1.09*
RC043	1	TBE	0	20	85	14.29	0.50	5.08	3.94	5.93	44	28	28	Clay Loam	0.79
RC043	2	TBE	20	26	84	10.79	0.29	3.14	2.01	6.09	40	38	22	Loam	1.12*
RC043	3	TBE	26	58	85	4.30	0.10	1.10	0.58	6.31	53	32	15	Sandy Loam	1.24*
RC043	4	TBE	58	70	76	3.54	0.06	0.72	0.34	6.42	62	29	9	Sandy Loam	1.37*
RC043	5	TBE	70	90	77	2.75	0.01	0.25	0.12	6.41	70	23	7	Sandy Loam	1.48*
RC056	1	TBE	0	30	87	13.70	0.35	3.75	2.74	6.16	61	22	17	Sandy Loam	0.86
RC056	2	TBE	30	75	83	7.19	0.25	2.86	2.04	6.15	61	22	17	Sandy Loam	1.12*
RC056	3	TBE	75	999	100	0.63	0.08	0.84	0.42	6.15	73	18	9	Sandy Loam	1.23*
RC060	1	SLU	0	30	90	13.78	0.22	2.13	1.71	6.34	40	35	25	Loam	1.30
RC060	2	SLU	30	60	92	16.01	0.07	0.49	0.36	7.58	28	36	36	Clay Loam	1.52
RC060	3	SLU	60	80	100	21.65	0.06	0.47	0.29	7.95	19	42	39	silty Clay Loam	1.59
RC067	1	TBE	20	35		17.65	0.34	3.81	3.35	7.09	44	35	21	Sandy Loam	1.01*
RC067	2	TBE	0	20		12.51	0.20	2.69	2.37	7.23	56	22	22	Loam	1.12*
RC067	3	TBE	95	999		15.08	0.18	3.07	2.64	7.24	66	19	15	Clay Loam	0.42*
RC070	1	HBE	0	9	97	35.50	0.88	9.63	8.98	6.24	28	38	34	Clay Loam	0.68*
RC070	2	HBE	9	25	99	24.35	0.45	4.63	3.63	6.63	38	33	29	Clay Loam	1.03*
RC070	3	HBE	25	55		8.73	0.11	1.06	0.52	6.37	51	34	15	Loam	1.25*
RC070	4	HBE	55	999		4.67	0.21	0.53	0.28	6.28	63	28	9	Sandy Loam	1.34*
RC076	1	SBE	0	27	100	19.54	0.56	4.85	4.35	6.43	14	50	36	Silty Clay Loam	0.94*
RC076	2	SBE	27	47	94	12.86	0.24	2.38	1.75	6.64	12	52	36	Silty Clay Loam	0.52*
RC076	3	SBE	47	63	100	9.89	0.16	1.55	0.84	6.52	20	55	25	Silt Loam	0.84*
RC091	1	TLU	0	11	86	12.50	0.45	4.28	3.82	5.28	47	26	27	Silty Clay Loam	1.04
RC091	2	TLU	11	23		10.61	0.21	1.79	1.55	5.95	55	23	22	Silty Clay Loam	1.19
RC091	3	TLU	23	40		7.44	0.08	0.62	0.49	6.48	48	25	27	Sandy Clay Loam	1.50
RC091	4	TLU	40	60	98	9.13	0.06	0.44	0.35	6.85	43	28	29	Clay Loam	1.49
RC092	1	TBE	0	12	90	12.18	0.52	4.81	4.08	5.11	26	36	38	Clay Loam	0.90
RC092	2	TBE	12	30	94	11.27	0.33	2.90	2.27	5.47	26	39	35	Clay Loam	1.17*
RC092	3	TBE	30	60	86	7.53	0.18	1.61	0.85	5.90	37	35	28	Clay Loam	1.23*
RC092	4	TBE	60	85	91	6.95	0.13	0.90	0.58	6.04	49	26	25	Sandy Clay Loam	1.23*
RC096	1	TSWG	0	13	100	10.70	0.34	3.45	2.91	5.70	27	41	32	Clay Loam	0.97

Profile Number	Horizon	Soil type	Start depth	End depth	Base Saturation (%)	Cation Exchange Capacity (cmol kg^{-1})	N (%)	C (%)	Corg (%)	pH	Sand 200-005mm % w/w	Silt 0002 - 005mm % w/w	Clay <0002mm % w/w	Textural Class	Bulk density g/cm3
RC096	2	TSWG	13	30	31	5.85	0.13	1.05	0.67	5.08	32	42	26	Loam	1.19
RC096	3	TSWG	30	70	37	5.69	0.09	0.35	0.25	5.11	19	46	35	Silty Clay Loam	1.2*
RC096	4	TSWG	70	90	42	3.67	0.07	0.28	0.20	5.12	30	41	29	Clay Loam	0.11*
RC096	5	TSWG	90	999	44	4.51	0.08	0.27	0.19	5.06	14	64	22	Silt Loam	1.41*
RC098	1	HBE	0	15	79	11.04	0.58	7.43	6.53	5.29	32	37	31	Clay Loam	0.78
RC098	2	HBE	15	25	76	6.84	0.26	3.29	2.62	5.24	35	40	25	Loam	1.07*
RC098	3	HBE	25	45	55	3.95	0.11	1.03	0.55	5.35	36	42	22	Loam	1.3*
RC098	4	HBE	45	90	66	3.08	0.10	0.53	0.30	5.43	42	36	22	Loam	1.32*
RC100	1	SLU	0	25	100	10.14	0.22	2.65	2.10	6.36	50	31	19	Loam	1.23
RC100	2	SLU	25	60	100	6.38	0.10	0.87	0.57	6.61	43	36	21	Loam	1.47
RC100	3	SLU	60	999	55	13.12	0.06	0.31	0.23	6.60	6	51	43	Silty Clay	1.47
RC105	1	HBE	0	26		17.97	0.44	7.26	6.64	5.98	44	37	19	Loam	0.86
RC105	2	HBE	26	38		13.23	0.18	4.45	4.01	6.30	47	38	15	Loam	0.96*
RC105	3	HBE	38	999	92	4.46	0.06	0.78	0.52	6.09	60	32	8	Sandy Loam	1.17*
RC107	1	SBE	0	20		11.03	0.31	2.95	2.33	6.11	41	35	24	Loam	1.12
RC107	2	SBE	20	35	86	8.12	0.19	1.97	1.40	5.57	37	39	24	Loam	1.11
RC107	3	SBE	35	60	94	5.83	0.08	1.11	0.54	6.01	48	38	14	Loam	0.83
RC107	4	SBE	60	90	60	3.69	0.11	1.69	0.82	5.92	49	41	10	Loam	0.99
RC107	5	SBE	90	120		1.11	0.03	0.21	0.13	6.14	50	32	18	Loam	1.47*
RC119	1	SBE	0	15	82	17.38	0.52	4.88	4.33	6.06	46	33	21	Loam	0.99
RC119	2	SBE	15	40	71	9.26	0.21	2.04	1.98	6.16	47	36	17	Loam	1.11
RC119	3	SBE	40	55	58	7.87	0.14	1.46	0.79	6.33	43	39	18	Loam	1.32*
RC119	4	SBE	55	85		1.20	0.04	0.40	0.23	6.51	54	33	13	Sandy Loam	1.42
RC119	5	SBE	85	120	59	2.69	0.03	0.16	0.10	6.73	59	28	13	Sandy Loam	1.53
RC123	1	TLU	0	15	89	15.12	0.31	2.85	2.44	6.96	32	43	25	Loam	1.11
RC123	2	TLU	15	35	88	14.59	0.31	2.85	2.65	6.88	29	40	31	Clay Loam	1.03
RC123	3	TLU	35	65	76	9.30	0.14	1.32	1.01	7.04	28	46	26	Loam	1.12
RC123	4	TLU	65	75	78	3.15	0.05	0.43	0.30	7.29	39	47	14	Loam	1.32*
RC123	5	TLU	75	110	74	3.75	0.05	0.28	0.17	7.40	38	33	29	Clay Loam	1.43*
RC129	1	HBE	0	25	94	16.34	0.42	5.60	4.93	5.66	38	38	24	Loam	0.81
RC129	2	HBE	25	58	100	6.07	0.11	1.37	0.91	6.15	43	36	21	Loam	1.10
RC129	3	HBE	58	85	100	3.40	0.06	0.76	0.44	6.07	51	31	18	Loam	1.31*
RC139	1	TSWG	0	23	99	2.55	0.18	1.98	1.50	5.18	62	29	9	Sandy Loam	1.27

Profile Number	Horizon	Soil type	Start depth	End depth	Base Saturation (%)	Cation Exchange Capacity (cmol kg^{-1})	N (%)	C (%)	Corg (%)	pH	Sand 200-005mm % w/w	Silt 0002 - 005mm % w/w	Clay <0002mm % w/w	Textural Class	Bulk density g/cm3
RC139	2	TSWG	23	45		0.79	0.02	0.21	0.16	6.60	56	36	8	Sandy Loam	1.76
RC139	3	TSWG	45	120		2.00	0.02	0.16	0.12	7.07	47	43	10	Loam	1.48*
RC142	1	SLU		23		10.41	0.24	3.30	2.49	6.21	69	24	7	Sandy Loam	0.97
RC142	2	SLU		50	79	4.21	0.07	0.93	0.42	6.07	55	34	11	Sandy Loam	1.16*
RC142	3	SLU		90		1.30	0.02	0.31	0.14	6.07	62	32	6	Sandy Loam	1.68*
RC143	1	TBE	0	20	81	6.51	0.26	2.81	2.30	5.68	56	33	11	Sandy Loam	1.02
RC143	2	TBE	20	35	74	5.59	0.14	1.55	0.90	6.23	54	33	13	Sandy Loam	1.15
RC143	3	TBE	35	60	55	3.72	0.11	1.16	0.49	5.85	58	34	8	Sandy Loam	1.06*
RC143	4	TBE	60	80	53	1.56	0.02	0.31	0.15	6.06	58	36	6	Sandy Loam	1.45*
RC143	5	TBE	80	100	99	0.94	0.01	0.20	0.11	6.05	49	43	8	Loam	1.5*
RC154	1	TBE	0	55	75	6.64	0.20	1.86	1.24	6.00	72	17	11	Sandy Loam	1.20
RC154	2	TBE	55	95	88	2.91	0.04	0.36	0.22	6.55	74	16	10	Sandy Loam	1.35
RC154	3	TBE	95	125		7.09	0.04	0.59	0.39	7.14	77	17	6	Loamy Sand	1.16
RC172	1	TLU	0	22	100	8.90	0.09	2.68	2.02	6.32	34	41	25	Loam	1.10
RC172	2	TLU	22	35	84	4.56	0.08	0.83	0.38	6.23	32	46	22	Loam	1.14
RC172	3	TLU	35	60	94	3.74	0.08	0.48	0.24	6.33	27	46	27	Clay Loam	1.33
RC172	4	TLU	60	85	99	3.65	0.08	0.37	0.19	6.35	66	21	13	Sandy Loam	1.34
RC172	5	TLU	85	100	100	3.25	0.37	0.18	0.10	6.30	35	42	23	Loam	1.6*
RC173	1	SBE	0	28	78	8.87	0.16	3.38	2.70	5.36	29	37	34	Clay Loam	1.11
RC173	2	SBE	28	45	96	6.97	0.16	1.10	0.89	5.98	31	41	28	Clay Loam	1.13*
RC173	3	SBE	45	65	96	4.51	0.15	1.23	0.73	5.95	53	23	24	Sandy Clay Loam	1.36*
RC173	4	SBE	65	70	94	5.34	0.13	0.61	0.31	5.92	25	46	29	Clay Loam	1.59*
RC182	1	SBE	0	15	91	13.66	0.54	6.16	5.23	5.61	44	26	30	Clay loam	0.74
RC182	2	SBE	15	25		7.60	0.31	3.57	2.57	5.90	47	28	25	Loam	0.89
RC182	3	SBE	25	42		2.23	0.15	1.67	0.72	5.97	55	30	15	Sandy loam	0.94
RC182	4	SBE	42	75	96	1.40	0.03	0.25	0.10	6.19	63	26	11	Sandy loam	1.49*
RC184	1	TSWG	0	12	95	10.89	0.62	6.74	5.48	5.52	41	26	33	Clay loam	0.86
RC184	2	TSWG	12	30	83	7.74	0.40	4.06	3.01	5.71	39	31	30	clay loam	1.01
RC184	3	TSWG	30	49	97	3.56	0.10	0.87	0.57	5.90	52	26	22	Sandy Clay Loam	1.17
RC184	4	TSWG	49	104	57	9.81	0.05	0.21	0.14	5.61	46	24	30	Sandy Clay Loam	1.66
RC186	1	TSWG	0	22	96	8.64	0.46	5.28	4.10	5.60	39	28	33	clay loam	0.96
RC186	2	TSWG	22	50	92	5.22	0.09	0.95	0.86	5.96	35	38	27	Clay Loam/Loam	1.49
RC186	3	TSWG	50	95		3.40	0.05	0.26	0.12	5.71	46	31	23	Loam	1.42

Profile Number	Horizon	Soil type	Start depth	End depth	Base Saturation (%)	Cation Exchange Capacity (cmol kg^{-1})	N (%)	C (%)	Corg (%)	pH	Sand 200-005mm % w/w	Silt 0002 - 005mm % w/w	Clay <0002mm % w/w	Textural Class	Bulk density g/cm3
RC186	4	TSWG	95	135	87	12.24	0.07	0.15	0.11	6.42	44	28	28	Clay Loam	1.57
RC187	1	HBE	0	21	100	19.05	0.64	6.67	6.12	6.44	58	26	16	Sandy Loam	0.63
RC187	2	HBE	21	32	100	6.07	0.15	1.53	0.92	6.44	27	64	9	Sandy Loam	0.58*
RC189	1	SBE	0	22	73	14.80	0.49	5.92	4.23	5.33	56	23	21	Sandy Clay Loam	0.78
RC189	2	SBE	22	37	85	9.92	0.27	3.20	2.62	5.44	58	24	18	Sandy Loam	1.07*
RC189	3	SBE	37	65		5.80	0.06	0.43	0.26	5.86	75	14	11	Sandy Loam	1.35*
RC215	1	TSWG	0	30	77	4.91	0.19	2.21	1.78	5.29	68	22	10	Sandy Loam	1.41
RC215	2	TSWG	30	48		3.54	0.12	0.87	0.66	6.02	59	26	15	Sandy Loam	1.52
RC215	3	TSWG	48	90		2.01	0.06	0.67	0.32	5.96	56	28	16	Sandy Loam	1.49
RC215	4	TSWG	90	115		0.89	0.02	0.16	0.07	5.99	75	14	11	Sandy Loam	1.73
RC220	1	HBE	0	23	92	15.36	0.63	7.63	6.40	5.51	35	30	35	Clay Loam	0.75
RC220	2	HBE	23	45	93	6.72	0.21	2.44	1.15	5.65	35	43	22	Loam	0.83
RC220	3	HBE	45	75	100	1.01	0.07	0.42	0.21	6.00	47	34	19	Loam	1.19
RC224	1	TSWG	48	125	74	6.51	0.60	6.20	5.31	5.16	20	40	40	Clay/Silty	1.48
RC224	2	TSWG	33	48	78	4.35	0.28	2.40	1.93	5.42	18	45	37	Silty Clay Loam	0.86
RC224	3	TSWG	0	10	44	5.98	0.16	0.98	0.61	5.52	29	42	29	Clay Loam	0.99
RC224	4	TSWG	10	33	47	4.19	0.08	0.27	0.16	5.59	46	36	18	Loam	0.09*

Figure SI3. Map of sites location through the Republic of Ireland. The map was created in ArcGIS v.10.2 (ESRI Inc., Redwoods, USA) and used to create the studied points map representation.(https://www.arcgis.com)

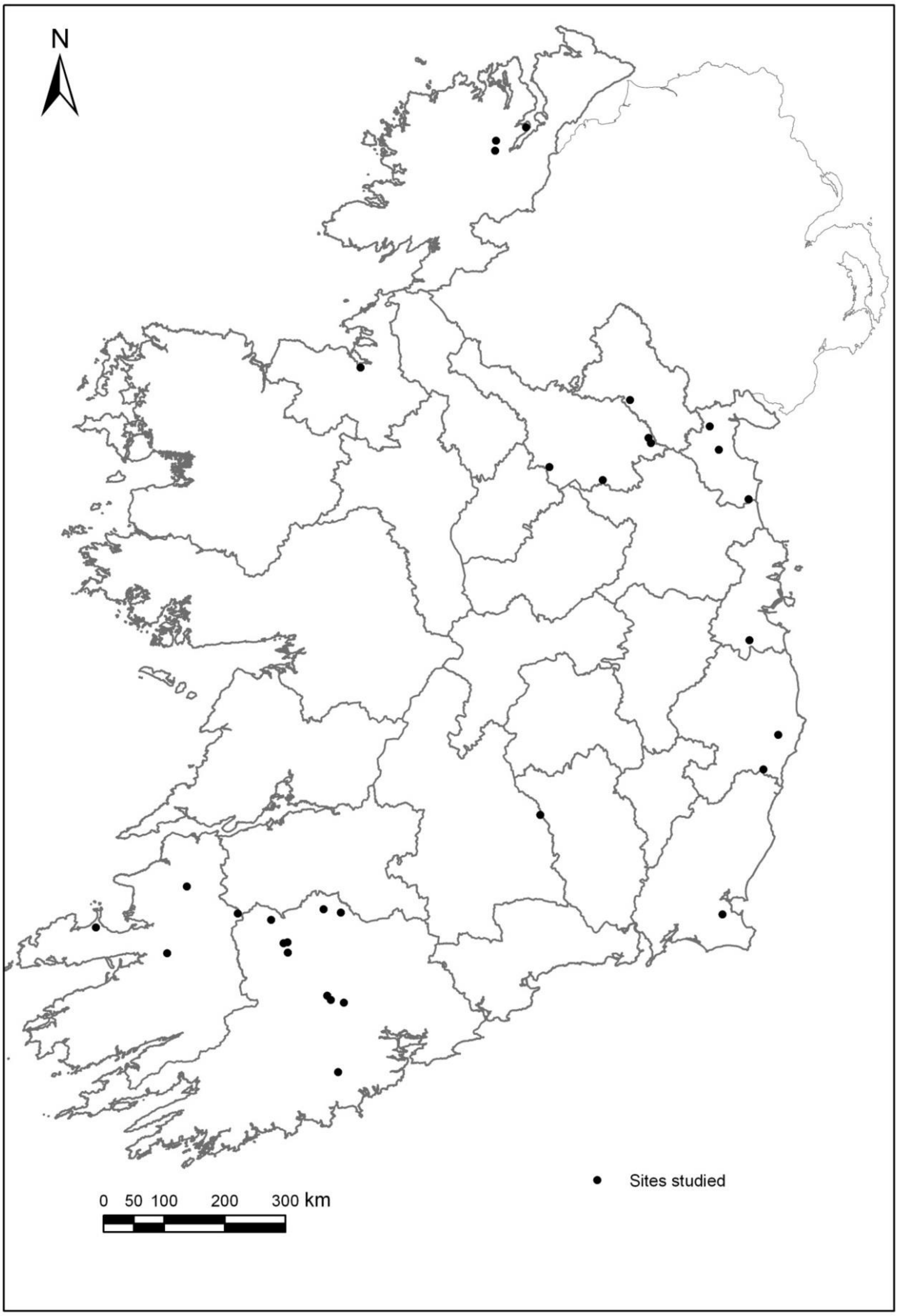

Supplement: Supplementary Information [file srep45635-s1.pdf]
